# Clitoral Hemangioma in Adults: A Case Report and Literature Review

**DOI:** 10.7759/cureus.68773

**Published:** 2024-09-06

**Authors:** Suhaila Al-Wahaibi, Manahil Mohammed

**Affiliations:** 1 Obstetrics and Gynaecology, University Medical City/Sultan Qaboos University Hospital, Muscat, OMN

**Keywords:** varicose veins, epidermal cyst, clitoral hemangioma, clitoral cyst, clitoral swelling

## Abstract

Hemangiomas are benign vascular tumors commonly seen in early childhood, typically occurring on the face, scalp, chest, or back. Clitoral hemangiomas, especially in adults, are exceptionally rare. This case report describes a unique presentation of clitoromegaly due to a cavernous hemangioma of the clitoris in a 39-year-old woman who presented with a progressive and worsening swelling of the clitoris for five years. A surgical excision of the cyst was performed, and a histological examination confirmed a cavernous hemangioma.

## Introduction

Hemangiomas are benign vascular tumors that arise from the proliferation of endothelial cells [1]. They are typically classified into two main types: infantile hemangiomas, which are the most common and usually appear shortly after birth, and congenital hemangiomas, which are fully developed at birth. These tumors are characterized by their rapid growth phase followed by a slower involution phase [2]. Hemangiomas predominantly affect the skin and mucous membranes and are most frequently observed on the face, scalp, chest, or back [1,3].

The occurrence of hemangiomas in the genital region is exceptionally rare, especially in adults. Among the limited documented cases, clitoral hemangiomas are notably uncommon [1,4]. The clitoris, being a highly vascular and sensitive structure, can present significant diagnostic and therapeutic challenges when affected by vascular anomalies.

Clitoromegaly, or the abnormal enlargement of the clitoris, can arise from various etiologies, including hormonal imbalances, congenital conditions, and neoplastic processes. Cavernous hemangiomas, a subtype of hemangiomas characterized by large, dilated vascular channels, can lead to clitoromegaly when they occur in the clitoral tissue. The rarity of this condition, particularly in adult women, underscores the importance of detailed case reports to enhance understanding and management strategies.

This report details a rare case of clitoromegaly due to a cavernous hemangioma of the clitoris in an adult female, providing insights into the clinical presentation, diagnostic workup, and therapeutic approach for this unusual vascular tumor.

## Case presentation

A 39-year-old woman presented to our urogynecology clinic with persistent clitoral swelling for five years, worsening over the last two years. She complained of painful swelling, aggravated by prolonged standing and more noticeable toward the end of the day. She experienced one episode of oozing from the swelling, which resolved spontaneously. The patient denied any recent trauma to the clitoral region and reported no sexual dysfunction.

Her obstetric history included three uncomplicated vaginal deliveries, the last occurring five years ago. She had undergone type 1 female genital mutilation as a child and a laparoscopic ovarian cystectomy for benign pathology. She had no significant past medical history.

**Figure 1:**
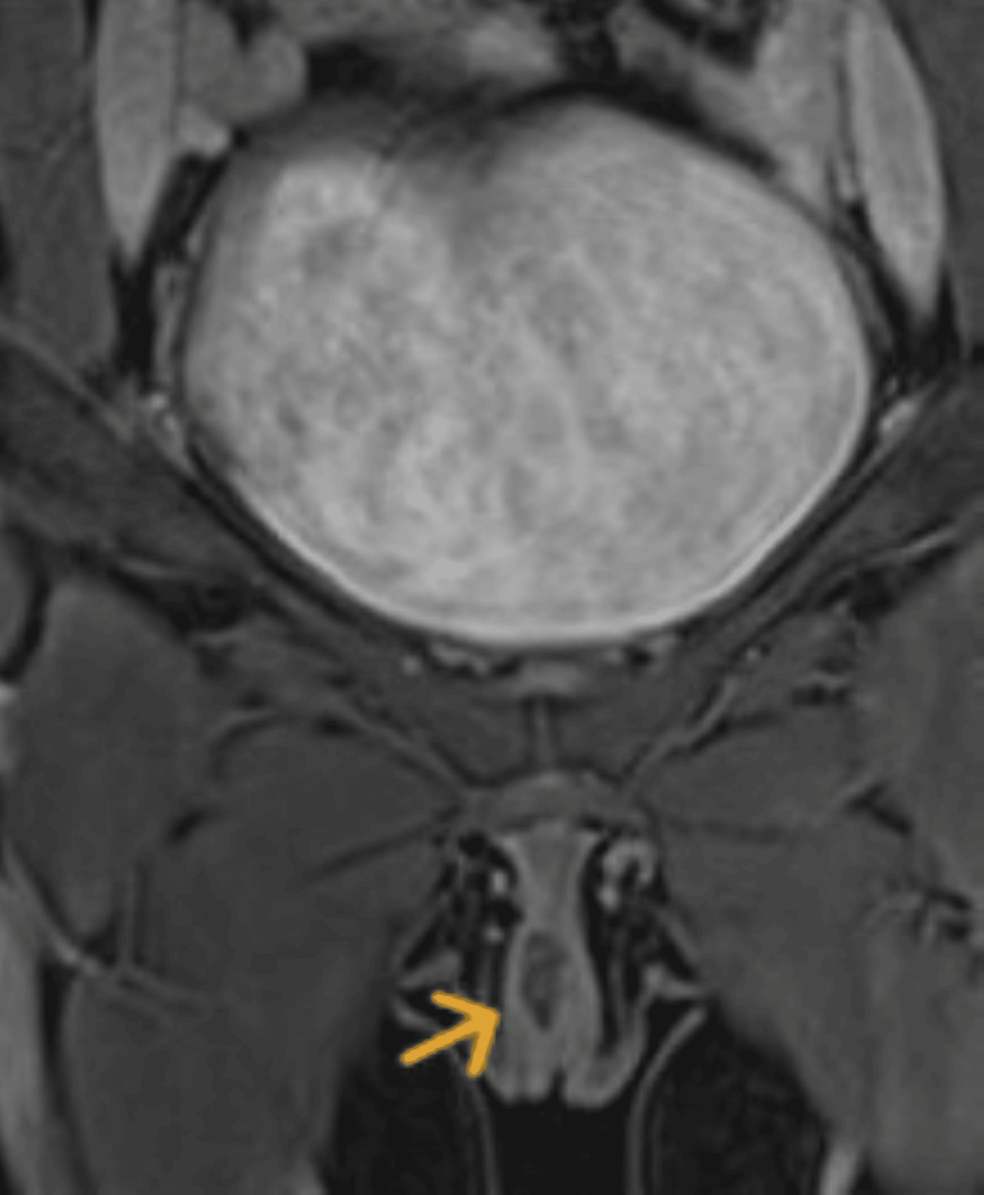
MRI showing a clitoral cyst.

Clinical examination revealed a 10 × 15 mm cystic mass at the clitoris, pink to bluish in color, with no discharge. The remainder of the pelvic examination was normal. An MRI of the pelvis showed an ovoid lesion near the clitoris, measuring 11 × 8 × 11 mm, with a high T2 signal and an isointense T1 signal. The lesion exhibited rim enhancement with intravenous contrast, without diffusion restriction, fat presence, or associated soft tissue or inflammatory changes (Figure 1).

**Figure 2:**
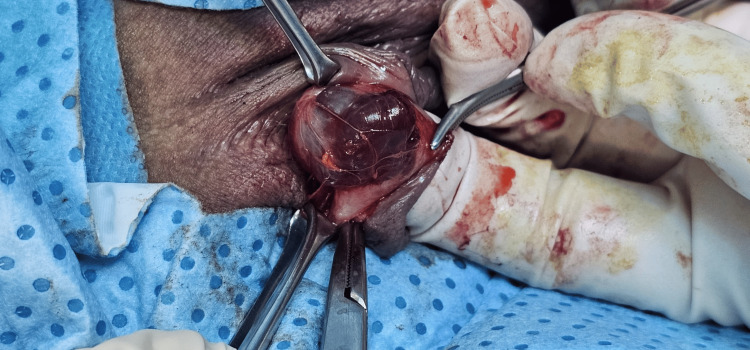
Intraoperative view of the hemangioma.

Despite the benign nature of the cyst, the patient was significantly bothered by it and opted for surgical management. She underwent cyst excision under general anesthesia. Intraoperative findings included a 1.5 × 1 cm cystic lesion at the prepuce of the clitoris. A 2 cm incision was made at the lateral border of the cyst, 2 cm away from the clitoral body to avoid injury. The cyst, adherent to the clitoral body and glans, was carefully dissected from superficial and deep tissues using scissors and an electrosurgical knife (Figure 2). The base was suture-ligated to maintain hemostasis. The provisional diagnoses included hemangioma, varicose veins, and endometrioma.

The operation lasted 60 minutes. The urinary Foley catheter was removed on the first postoperative day, and the patient was discharged. Follow-up visits at two weeks and two months showed complete recovery with no signs of recurrence. The patient reported no changes in sensation at the clitoris or external genitalia. Histological examination confirmed the diagnosis of a cavernous hemangioma.

## Discussion

Hemangiomas typically manifest at birth or during early childhood. The occurrence of clitoral hemangiomas in adults is highly unusual [2,5]. Clinical presentations often include painless, progressive enlargement of the clitoris, though patients may also experience discomfort, bleeding, or interference with sexual function.

Diagnosis relies on clinical examination and imaging studies. Doppler ultrasonography is a valuable non-invasive tool for assessing the vascular nature of the lesion [1,6]. MRI provides detailed insights into the extent and characteristics of the hemangioma, distinguishing it from other vascular malformations or neoplastic conditions. Unfortunately, as in our case, MRI was not able to confirm the diagnosis of hemangioma. Histopathological examination remains the definitive diagnostic method, revealing proliferating blood vessels lined by endothelial cells.

Although hemangiomas are commonly occurring neoplasms, clitoral cavernous hemangiomas are extremely rare, with only six cases documented in the English literature. Most of these cases were identified in young and adolescent patients, and only one was diagnosed using MRI before pathological examination (Table 1).

**Table 1:** Details of the clitoral hemangioma cases reported so far. Adapted from Geramizadeh et al. [4].

Reported case	Age (years)	Presumptive diagnosis	Pathological diagnosis
Haritharan et al. [8]	5	Upper external genitalia mass	Solitary vascular malformation
Kajal et al. [1]	6	Vascular swelling of the clitoris	Cavernous hemangioma
Nayyar et al. [3]	10	Clitoral mass	Cavernous hemangioma
Germaizadeh et al. [4]	16	Clitoral hypertrophy	Cavernous hemangioma
Kaufmann-Freidman [5]	18	Adrenogenital syndrome	Cavernous hemangioma
Bruni et al. [6]	20	Clitoral mass	Cavernous hemangioma
Current case	39	Clitoral mass	Cavernous hemangioma

Management strategies for clitoral hemangiomas depend on the lesion’s size, symptoms, and patient preferences. Asymptomatic and small hemangiomas may be managed conservatively with regular follow-up. Interventional treatments for symptomatic lesions include surgical excision, complete removal is the most definitive treatment for symptomatic or complicated hemangiomas, with generally favorable outcomes and minimal recurrence. A possible management option is laser therapy; pulsed dye laser therapy targets the vascular components of hemangiomas, causing regression [7]. Pharmacotherapy is also an option using systemic treatments, such as beta-blockers like propranolol [1,2]. Both laser and pharmacotherapy have shown efficacy in treating hemangiomas in other parts of the body, though their application for clitoral hemangiomas is not well-documented and requires further research [1,7].

With appropriate treatment, the prognosis for clitoral hemangiomas is generally positive. Surgical excision tends to yield excellent results with a low risk of recurrence [3-6,8]. Complications are rare but can include infection, scarring, or recurrence. Early diagnosis and intervention are critical to preventing complications and ensuring favorable outcomes. Continuous follow-up is essential to monitor for potential recurrence or the development of new symptoms.

## Conclusions

Clitoral hemangiomas in adults are rare and present unique diagnostic and management challenges. Accurate diagnosis involves clinical examination, imaging, and histopathology. Having a high index of suspicion for such lesions is crucial in counseling and planning surgical management. Treatment options range from conservative management to surgical excision and laser therapy, with generally favorable outcomes with surgical excision. Further research is necessary to establish standardized management guidelines for this rare condition in the adult population.
